# Pyriform Sinus Fistula in Children: Preferred Imaging Modality and Risk Factors for Diagnostic Delay

**DOI:** 10.3389/fped.2020.575812

**Published:** 2020-10-30

**Authors:** Tong Chen, Guijie Ge, Jianglong Chen, Xiuhao Zhao, Qingfeng Sheng, Linlin Zhu, Weijue Xu, Jiangbin Liu, Zhibao Lv

**Affiliations:** Department of General Surgery, Shanghai Children's Hospital, Shanghai Jiao Tong University, Shanghai, China

**Keywords:** barium esophagography, computed tomography, diagnostic delay, inflammation, pyriform sinus fistula

## Abstract

**Background:** Diagnostic delay of pyriform sinus fistula (PSF) continues to challenge clinicians, and the preferred imaging modality is yet to be verified. The purpose of this study was to investigate the preferred imaging modality for PSF and the possible risk factors for a longer diagnostic delay.

**Methods:** Medical records of patients with a surgically confirmed PSF from 2014 to 2018 were retrospectively evaluated. A comparison of the first esophagography timing with a true-positive (TP) result and that with a false-negative (FN) result was made. Data of computed tomography (CT) performed immediately after esophagography were also analyzed. In addition, the factors related to diagnostic delay were analyzed using multivariate regression models.

**Results:** A total of 147 patients ranging in age from 0 to 16 years (median: 5.2 years) were included. The mean time since the symptom onset of the first esophagography with TP result was significantly longer than that of the examination with FN result (95.18 ± 79.12 vs. 52.59 ± 42.40 days, *P* = 0.032). When the time since the symptom onset was less than 12 weeks, the false-negative rate (FNR) of the first esophagography was declining dramatically with a longer time interval. Among 18 cases with an FN result of the first esophagography, the fistulous tract was finally identified in seven cases using an immediate CT. The mean of diagnostic delay was 12.28 months. Besides, rural residency was an independent risk factor for a longer diagnostic delay.

**Conclusion:** Joint examination of esophagography and an immediate CT is the preferred imaging modality for the diagnosis of PSF in children. It is inadvisable to perform the first esophagography when the time since the symptom onset is less than 12 weeks. Besides, the rural residency is an independent risk factor for a longer diagnostic delay.

## Introduction

First reported by Raven (1), pyriform sinus fistula (PSF) serves as a rare congenital anomaly due to incomplete obliteration of the third or fourth branchial pouch (2). Approximately 80% of the patients with PSF have their onset in childhood (3). PSF often presents as recurrent flu-like symptoms followed by neck infections, especially on the left side (4). Due to the rarity, PSF is frequently neglected in the differential diagnosis, which in turn results in a diagnostic delay. Without the definitive treatment, neck infections may recur in 89–94% of PSF patients (5, 6). More severely, recurrent neck infections may give rise to the fibrosis and scarring, making it technically notorious for surgeons to perform a fistulectomy. Indeed, a recent study demonstrated that the history of neck infection is a risk factor for recurrence after fistulectomy (7). If not treated properly, severe neck infections attributable to PSF can lead to serious complications and even death (8).

For the diagnosis of PSF, both barium esophagography and laryngoscopy are considered to be the most effective modalities (5, 6, 9, 10). Without the need for general anesthesia, esophagography is more convenient to perform and more widely accepted, but may potentially yield false-negative (FN) result if performed during the acute infection phase (11). On the other hand, children with PSF cannot wait too long because neck infection may recur in the following weeks (12, 13). However, the timing of esophagography since symptom onset remains unclear. In addition, one case report revealed that PSF in a child showing a negative result of esophagography and the fistulous tract was finally identified using computed tomography (CT) performed immediately after esophagography (14). In order to explore the preferred imaging modality for PSF and the risk factors related to diagnostic delay, 147 patients with surgically confirmed PSF were included in this retrospective study.

## Materials and Methods

### Patient Enrollment

**Figure 1:**
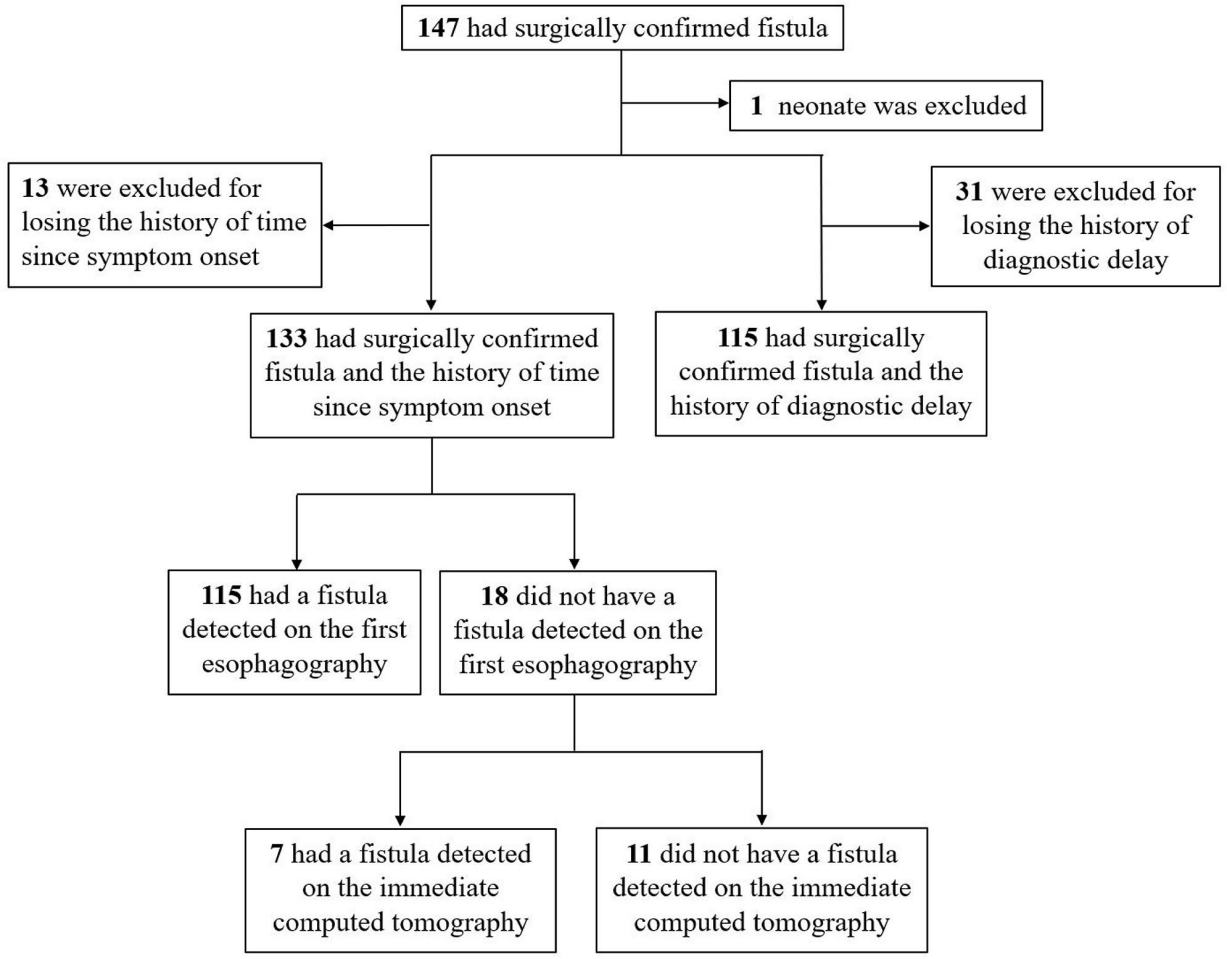
Flowchart indicating the inclusion of patients.

This study included children with a surgically confirmed PSF who were admitted to the general surgery department of our hospital between January 2014 and December 2018. Medical records of imaging tests for the diagnosis of PSF were retrospectively reviewed. Figure 1 shows the flowchart of patient selection process.

### Imaging

All patients with suspected PSF were imaged preoperatively using barium esophagography. Standard esophagography technique was selected. The imaging was performed using one device (Uni-vision). Barium sulfate was mixed with sterile water to prepare a dilute solution (300–400 g/L). Most children were able to swallow the dilute solution in the upright position. Otherwise, he/she would be arranged in a supine position and swallow the dilute solution from a nursing bottle. Immediately after esophagography, CT was routinely performed from the pharynx oralis to the suprasternal fossa. Specifically, a GE Light Speed VCT with a 3-mm reconstructed slice thickness was used. If the child does not cooperate with the procedures, he/she would be sedated using oral chloral hydrate (0.5 ml/kg). For contrast enhancement, an iodinated contrast agent (2 ml/kg) was injected intravenously at 2 ml/s. Coronal and sagittal reconstruction images were obtained. Esophagography and CT were performed by two experienced pediatric-certified radiologists who both possessed at least a 5-year clinical experience.

### Timing of the First Esophagography and Diagnostic Delay

Neck swelling or mass, sore throat, and fever were the first occurred symptoms of PSF. Timing of the first esophagography was defined as the interval between the symptom onset date of the last inflammatory episode and the date of undergoing the examination. Besides, diagnostic delay was defined as the interval between the onset date of the first occurred symptoms and the date of imaging diagnosis as PSF.

### Assessment of Surgically Confirmed PSF

The endoscopic-assisted surgery was performed by a surgeon with a 30-year clinical experience and his assistants. In order to expose the fistulous tract, inflammatory and fibrotic tissues surrounding the inferior cornu of the thyroid cartilage were resected. One of the assistants conducted the endoscopy (model GIF-160 endoscope; Olympus, Tokyo, Japan). Light was used as an indicator to the operation field. The internal orifice was found in the pyriform fossa, and a 3 French catheter was then inserted into the fistulous tract. In cases with the catheter slipping out of the loci, methylene blue (0.5–1.0 ml) was injected into the sinus as an indicator. Under this condition, PSF was deemed to be confirmed by the surgery. Subsequently, the fistulous tract was ligated and excised as high as possible. If the thyroid gland was involved, partial thyroidectomy would be performed. The fistulous tract and surrounding fibrotic tissues with or without one part of the thyroid were excised *en bloc*.

### Statistical Analysis

All statistical analyses were carried out using the Statistical Package for the Social Sciences software (version 22.0, Chicago, USA). For continuous variables, the results were recorded as mean with standard deviation (SD) or median with interquartile range (IQR). Categorical variables were described as N with percentage, and confidence intervals (CIs) were expressed as 95% CI. The false-negative rate (FNR) was computed as the number of FN results/(the number of true-positive results + the number of FN results). Student's *t*-test was utilized to compare the parametric continuous variables, whereas the Wilcoxon unpaired test was utilized to assess the non-parametric continuous variables. Linear regression models with the log-transformation of diagnostic delay were used. Then, we derived the difference in log-time between categories of patients and its 95% CI. Non-colinear variables with *P* < 0.2 in the univariate regression analysis were included in the multivariate regression models. Two-sided confidence level of *P* < 0.05 indicated significance.

## Results

### Patient Characteristics

One hundred and forty-seven patients aged 0 to 16 years (median: 5.2 years) were included. The sociodemographic characteristics of the patients are depicted in Table 1. No gender predominance was found, and 93.20% of the lesions were on the left side. Eighty-five patients (57.82%) were initially misdiagnosed before consultation at our hospital. Ultrasonography (US) was performed in 76 patients, and the fistulous tract was detected in 32 patients (42.11%). Four patients underwent magnetic resonance imaging (MRI), which yielded a positive result in only one case (25%).

**Table 1:** Sociodemographic characteristics of patients with surgically confirmed fistula.

**Characteristics**	***N***	**%**
Sex (*n* = 147)		
Female	73	49.66
Male	74	50.34
Age at diagnosis (*n* = 147)		
≤ 1	9	6.12
>1, ≤ 3	23	15.65
>3, ≤ 7	62	42.18
>7	53	36.05
Laterality (*n* = 147)		
Left	137	93.20
Right	9	6.12
Bilateral	1	0.68
Ethnic group (*n* = 147)		
Han Chinese	146	99.32
Hui Chinese	1	0.68
Gestational age (*n* = 147)		
Preterm* individuals	3	2.04
Term-born individuals	144	97.96
Delivery mode (*n* = 142)		
Eutocia	78	54.93
Cesarean	64	45.07
Birth weight (*n* = 135)		
Low birth weight	5	3.70
Normal birth weight	119	88.15
High birth weight	11	8.15
Place of residence (*n* = 144)		
Urban	60	41.67
Town	52	36.11
Rural	32	22.22

* *Preterm refers to the baby born alive before 37 weeks of pregnancy*.

### Preferred Imaging Modality

**Figure 2:**
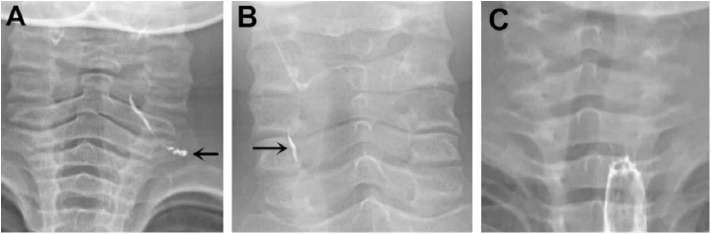
Barium esophagography indicating pyriform sinus fistula (PSF). **(A)** PSF was detected on the left side of a 4-year-old boy on the first esophagography, which was conducted 132 days after symptom onset. **(B)** PSF was detected on the right side of a 9-year-old girl on the first esophagography, which was conducted 151 days after symptom onset. **(C)** A 2-year-old girl had surgically confirmed PSF on the left side, but no fistula tract was detected on the first esophagography, which was conducted 22 days after symptom onset. PSF, pyriform sinus fistula.

**Figure 3:**
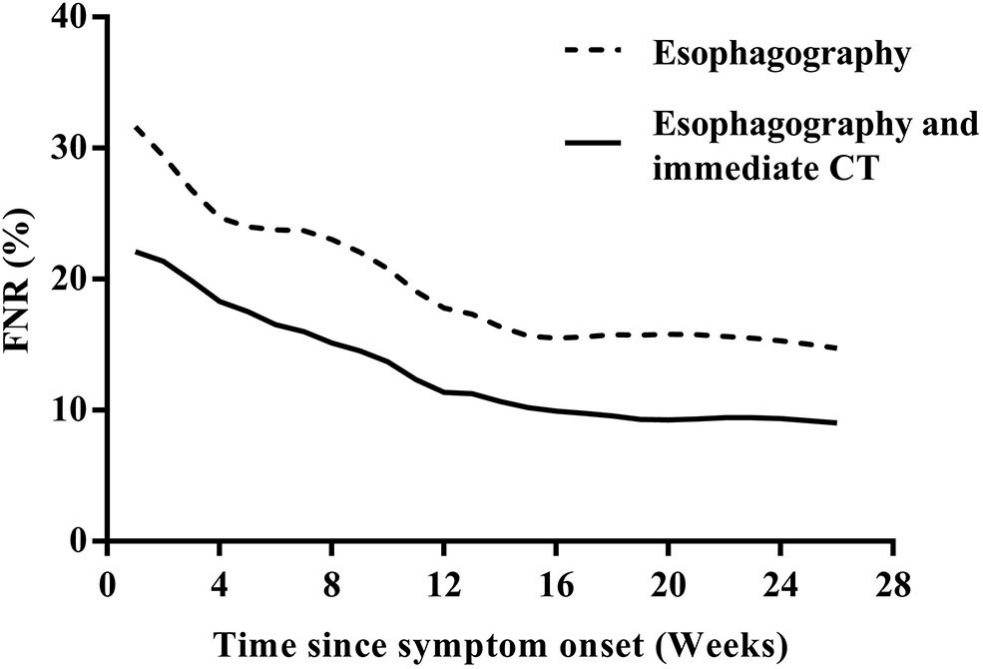
The FNR curves of the first esophagography and the first esophagography with immediate CT according to the time since the symptom onset. CT, computed tomography; FNR, false-negative rate.

Among the 133 children with a history of the first esophagography timing, the fistulous tract was identified in 115 children (86.47%) using the first esophagography. Images of barium esophagography indicating PSF are shown in Figure 2. The comparison of the inflammation-related presentations between the first esophagography with TP result and the examination with FN result is listed in Table 2. Timing of the first esophagography with TP result was significantly longer than that of the examination with the FN result (95.18 ± 79.12 vs. 52.59 ± 42.40 days, *P* = 0.032). Imaging tests with FN result according to time since symptom onset are listed in Table 3 (*P* = 0.041). The FNR was 23.8% when the first esophagography was performed less than 1 month since symptom onset. In contrast, The FNR was 7.4% when the first esophagography was performed more than 12 weeks since symptom onset. When the time since symptom onset was within 12 weeks, the FNR of the first esophagography was declining dramatically with a longer time interval (Figure 3). Among the 18 children with an FN result of the first esophagography, the fistulous tract was identified in seven cases (38.89%) using an immediate CT. Among the 11 cases with FN results on both the first esophagography and an immediate CT, the fistulous tract was finally identified using esophagography during a quiescent period.

**Table 2:** Differences of inflammation-related presentations between the first esophagography with TP result and the examination with FN result.

**Variable**	**TP (*n* = 115)**	**FN (*n* = 18)**	*P*-value*
Time since symptom onset (d) Mean ± SD (range)	95.18 ± 79.12 (1-652)	52.59 ± 42.40 (1-151)	0.032
Number of inflammatory episodes before esophagography Mean ± SD (range)	3.28 ± 2.60 (10-16)	2.61 ± 0.98 (1–4)	0.285
Times of I&D before esophagography Mean (95% CI)	1.63 (1.21–2.03)	1.05 (0.43–1.68)	0.292

* *Compared the value between the first esophagography with TP results and the examination with FN results. CI, confidence interval; FN, false negative; I&D, incision and drainage; SD, standard deviation; TP, true positive*.

**Table 3:** Imaging tests with FN result according to the time since symptom onset.

**Variable**	**Time since symptom onset (m)**				*P*-value*
	**≤1**	**>1, ≤2**	**>2, ≤3**	**>3**	
Number of patients	21	22	36	54	
Esophagography with FN results, n (%)	5 (23.81)	6 (27.27)	3 (8.33)	4 (7.41)	0.041
Esophagography and immediate CT with FN results, n (%)	4 (19.05)	3 (13.64)	2 (5.55)	2 (3.70)	0.096

* *Compared the value among groups. Data are given as the number of patients unless otherwise indicated. CT, computed tomography; FN, false negative*.

### Diagnostic Delay

To explore the predictive factors of diagnostic delay, 32 patients with no history of diagnostic delay were excluded (Figure 1). The mean of the diagnostic delay was 12.28 months. In 47 cases (40.87%), the diagnostic delay was over 6 months; in 33 cases (28.70%), it was over 12 months; and in 15 cases (13.04%), it was over 3 years. The univariate and multivariate regression analyses of possible factors for a longer diagnostic delay are listed in Table 4. According to the multivariable analysis, rural residency was an independent risk factor significantly correlated with a longer diagnostic delay.

**Table 4:** Univariate and multivariate regression analyses for the potential characteristics affecting diagnostic delay of PSF.

**Diagnostic delay**		**Univariate analysis**		**Multivariate analysis**	
**Variable**	**Median (d; 25–75th)**	**Difference in log (95% CI)**	*P*-value*	**Difference in log (95% CI)**	*P*-value*
**Gestational age**					
Preterm individuals	10 (2-10)	0		0	
Term-born individuals	90 (30-365)	−0.214 (−4.354–−0.343)	0.022	−0.173 (−3.652–−0.059)	0.043
**Age at first symptom onset (year)**					
≤ 1	875 (205–2445)	0	<0.001	0	
>1, ≤ 3	210 (75–1138)	−0.157 (−2.910–1.219)	0.408	−0.029 (−2.103–1.799)	0.873
>3, ≤ 7	120 (30-365)	−0.290 (−1.616–−0.043)	0.039	−0.253 (−1.504–0.120)	0.093
>7	30 (12-128)	−0.205 (−1.381–0.280)	0.060	−0.215 (−1.031–0.115)	0.113
**Place of residence**					
Urban	30 (10-180)	0	<0.001	0	
Town	135 (38–365)	0.350 (0.493–1.801)	0.001	0.297 (0.318–1.608)	0.004
Rural	730 (68–1460)	0.534 (0.687–1.511)	<0.001	0.502 (0.598–1.430)	<0.001

* *Compared the value among groups. PSF, pyriform sinus fistula*.

## Discussion

Collectively, our data demonstrated that when the time since symptom onset was less than 12 weeks, the FNR of the first esophagography was declining dramatically with a longer time interval. In some patients with an FN result of esophagography, the fistulous tract could be identified using an immediate CT. In addition, the rural residency served as an independent risk factor affecting the diagnostic delay of PSF.

The recommended imaging test for neonates with suspected PSF is CT/MRI (14, 15), so we excluded PSF neonate from the pool of cases to investigate the preferred imaging modality for PSF in children (Figure 1). Although barium esophagography is widely accepted as a safe and reliable option for the diagnosis of PSF, it should be noted that barium esophagography serves as a technically demanding modality (16), and an experienced radiologist in esophageal fluoroscopy is important for an accurate diagnosis in PSF. This may explain why the true-positive rate of barium esophagography for the diagnosis of PSF was reported to be not high in a minority of previous studies (11, 17). Consistent with most other previous studies (5, 6, 18–20), our data demonstrated that among the 133 children with a history of the first esophagography timing, the fistulous tract was identified in 115 children (86.47%) by the first esophagography. When the time since symptom onset was more than 12 weeks, the true-positive rate of the first esophagography was as high as 92.6% (50/54). If esophagography or CT is justified by medical need, the risk of radiation exposure will be relatively negligible concerning the diagnostic information obtained (21). On the other hand, radiation exposure was reported to be associated with an increased risk of cancer in children (22). Thus, the FNR of radiation-related imaging tests should be controlled at low levels if possible. Our results demonstrated that the timing of the first esophagography with TP result was significantly longer than that of the examination with the FN result. During the acute infection phase, the edematous wall of the fistulous tract might prevent the barium sulfate from passing smoothly. When esophagography was performed less than 12 weeks since the symptom onset, the FNR was declining dramatically with a longer time interval, and early esophagography was, therefore, inadvisable. Theoretically, scarring attributable to recurrent infections could make it less difficult for the barium to enter the fistula orifice and retain in the fistulous tract (14). Despite this, our data revealed that there was no significant difference between the number of inflammatory episodes before esophagography with TP result and that of the examination with FN result. In some patients with an FN result of esophagography, the fistulous tract was identified using an immediate CT. In addition, CT is able to delineate the anatomical path of the fistulous tract, identify whether the thyroid gland is involved, and exclude other anomalies of the neck (23–25). With the joint examination of esophagography and an immediate CT, the FNR of imaging tests could be controlled at an acceptable level.

PSF is one of the most underreported disease in children, and the diagnostic delay is frequently reported (26–28). In this study, the mean diagnostic delay was 12.28 months. Similar to our results, the mean diagnostic delay in the literature ranged from 6 to 18 months (7, 28–30). Overall, the diagnostic delay could be categorized as patients' delay in seeking medical care and doctors' delay in making an accurate diagnosis. Based on the multivariate analysis, it was found for the first time that rural residency was an independent risk factor for a longer diagnostic delay in PSF. The reasons for this finding might be multifaceted. Rural areas are characterized by a low population density and large geographic regions. Children with PSF living outside urban areas have less access to diagnostic and therapeutic services, higher costs, and longer travel distances to achieve medical care. It remains a challenge for the health care system to improve the access to medical care across different demographic regions.

The present study has several limitations. First, this was a retrospective and single-institution study. In addition, the overall diagnostic delay was not categorized as patient-dependent and doctor-dependent delays to further the understanding of diagnostic dilemma.

In conclusion, joint examination of esophagography and an immediate CT is the preferred imaging modality for the diagnosis of PSF in children. It is inadvisable to perform the first esophagography when the time since the symptom onset is less than 12 weeks. Furthermore, rural residency is an independent risk factor for a longer diagnostic delay in PSF.

## Data Availability Statement

All datasets generated for this study are included in the article/supplementary material.

## Ethics Statement

The studies involving human participants were reviewed and approved by Medical research ethics committee of Shanghai Children's Hospital, Shanghai Jiao Tong University. Written informed consent to participate in this study was provided by the participants' legal guardian/next of kin. Written informed consent was obtained from the minor(s)' legal guardian/next of kin for the publication of any potentially identifiable images or data included in this article.

## Author Contributions

TC and ZL were responsible for the study design. TC, GG, and JC collected and analyzed the data. TC, GG, XZ, QS, LZ, WX, and JL wrote the manuscript. All authors contributed to the article and approved the submitted version.

## Conflict of Interest

The authors declare that the research was conducted in the absence of any commercial or financial relationships that could be construed as a potential conflict of interest.
